# How to treat infections in a surgical intensive care unit

**DOI:** 10.1186/1471-2334-14-193

**Published:** 2014-11-28

**Authors:** Jan De Waele, Liesbet De Bus

**Affiliations:** Department of Critical Care Medicine, Ghent University Hospital, De Pintelaan 185, 9000 Gent, Belgium

## Abstract

The management of infections in surgical intensive care unit patients poses specific challenges. Although the overall approach to the patient is no different from other patients, diagnosis is often problematic. As in other infections, multidrug resistance is increasingly described, and changes in pharmacokinetics may require different dosing strategies. Also the need for source control adds a level of complexity to the management of the patient. Whereas source control was a purely surgical issue before, percutaneous drainage has emerged as an important alternative. Appropriate timing of source control often remains difficult to determine, but in most severe infections source control should not be delayed. But also the need for a multidisciplinary approach can make the decision making difficult. New concepts such as dedicated source control teams may further assist in selecting the most appropriate treatment strategy and further improve outcome of surgical severe sepsis patients.

## Review

### Introduction

Severe infections in surgical patients may be the reason for admission in some, but may also develop during intensive care unit (ICU) stay in others. These infections are an important burden in modern critical care, in terms of morbidity, mortality and resource use. In a recent study from China, mortality was high in surgical ICU (SICU) patients developing severe sepsis (48.7%) [1] and nursing workload high.

Abdominal infections more in particular, are associated with a long ICU stay, more shock and acute kidney injury and a higher mortality compared to other infections [2, 3] and therefore deserve proper attention.

Although there are no standardized definitions of what constitutes SICU patients, patients admitted after recent surgery (within the preceding 2-4 weeks, including emergency surgery (a non-scheduled operation within 24 hours of the onset of symptoms or injury)) are considered surgical [3, 4] and the focus of the current review.

The overall approach to infections in surgical patients is comparable to other patient categories, with rapid administration of appropriate antibiotics as one of the most important elements. The role of source control however should not be underestimated and is to be considered more often. To this extent, communication and interaction with other specialties such as surgery and interventional radiology is pivotal and preferably these patients should be managed in a multidisciplinary way. Diagnosis, both of primary infections or infections where the initial therapy has failed are particularly challenging but also the application of other, newer concepts such as antimicrobial de-escalation may be different.

Rather than listing antibiotic therapy schemes for commonly encountered infections, we will review specific aspects of the treatment of infections in SICU patients.

### Epidemiology of infections in surgical ICU patients

SICU patients apparently are at the highest risk to be diagnosed with an infection, presumably because the infection itself was the cause for admission to the ICU more often; as an example, in the Sepsis Occurrence in Acutely ill Patients (SOAP) study, 89% of the abdominal infections were non-ICU acquired [3], the highest percentage of frequently encountered infections studied.

In the European Prevalence of Infections in Intensive Care (EPIC)-II study, about two thirds of the infected patients were considered surgical patients (emergency surgery mostly, but also trauma and elective surgery) [4]. Obviously not all of these patients had typical surgical sources of infection.

In a large study from China, abdominal infections accounted for 72 percent of the infections in SICU patients diagnosed with severe sepsis, with acute pancreatitis and gastrointestinal perforation as the leading sources of infection [1]. Notably more than half of the cases had infections in multiple locations.

As the sources of infections are different compared to general ICU patients, these SICU infections also have a distinct microbiology pattern. Cheng et al. found that 43.7 percent of infections were polymicrobial, with a comparable contribution of Gram-positive and Gram-negative bacteria [1]. Fungi were also present in a considerable number of infections in this multicenter study (28.3%). In the SOAP study, Gram-positive bacteria (mainly *Streptococcus* D) and *Escherichia coli* were more frequently isolated in surgical patients [3].

### Diagnosing infections in surgical ICU patients

Typically, SICU patients with infectious complications are either admitted with an infection (mostly postoperative) or they develop it during their admission for another primary diagnosis. Both categories pose specific problems in terms of timely diagnosis to allow early therapy.

Diagnostics of infections in SICU patients admitted for non-infectious reasons can be puzzling as the tools we tend to rely on are unreliable in many situations. SIRS criteria are non-specific and frequently a reflection of postoperative inflammation, trauma, burns or any other inflammatory process. Similarly, conventional biomarkers of inflammation are often useless to diagnose infections immediately after another event. Also signs of impending organ dysfunction in a setting of severe sepsis, such as hypotension or oliguria, may be the result of other postoperative complications such as bleeding, fluid losses or under-resuscitation.

In patients admitted after surgical (or interventional) treatment, the diagnosis of recurrent infection due to failed source control is a problem that poses particular challenges. In a prospective multicenter study, Van Ruler et al. found that – contrary to what one may think – the extent of peritonitis, the source of the infection, the type of contamination or operative variables such as the presence of an anastomosis, were not associated with recurrent infection. Adding postoperative symptoms such as fever and parameters of organ dysfunction to the multivariate model, could identify patients requiring additional source control measures [5]. Most commonly used scoring systems perform poorly in this setting, only acute organ dysfunction scores such as the SOFA score were somewhat useful [6], although the AUROC was only 0.61 for the best discriminative score. Biomarkers may be superior in identifying patients without surgical source control; in a small study Novotny et al. found that a ratio of PCT on day 2 to PCT on day 1 of 1.03 or higher could discriminate failed from effective source control [7].

Clinical suspicion is probably best to track failed source control early; when available, PCT can be used for confirmation, but further work up remains necessary. Bedside ultrasound and abdominal CT scan represent the best tools; ultrasound has the advantage that it is readily available in most units and does not require transportation to the CT lounge and contrast administration. Both imaging techniques can be complemented by fine needle aspiration of suspected collections, or percutaneous drainage (PCD) in case of collections amenable to catheter treatment. In most situations, PCD is preferable over open surgery to confirm the diagnosis.

### Antibiotic therapy

Similar to the general ICU population, the empirical antibiotic scheme should cover the probable pathogen(s). This knowledge needs to be supplemented with local ecology data to determine the most appropriate empirical antimicrobial regimen. In abdominal infections inadequate antimicrobial therapy is associated with an increase in mortality rates in several studies [8–10].

As in other infections involvement of multidrug resistant (MDR) pathogens is a major concern. Seguin et al. found this risk to be particularly elevated in patients who were hospitalized for 5 days or longer and after previous exposure to antibiotics; when both criteria were present MDR was present in 38% of the infections compared to 2% when both were absent [11]. Swenson et al. reported an association between health-care exposure; e.g. current ICU admission, hospitalization for more than one week, but also including hospitalization within one month prior to the infection, residence in a nursing home or rehabilitation facility, and the occurrence of MDR pathogens [12].

When selecting empirical and directed antimicrobial therapy in the setting of surgical infections, we have to take some limitations of the microbiology diagnostic techniques into account. Abdominal infections are typically polymicrobial with both Gram-positive and Gram-negative, aerobe and anaerobe bacteria contributing in most patients. Depending on the techniques used, up to 10-15 microorganisms can be found in cultures from intra-abdominal infections, but pathogenicity may be difficult to assess. Anaerobe microbes are difficult to culture and even if these are not reported by the microbiology lab, these should be covered by the antibiotic therapy [13]. This is equally relevant in necrotizing skin and soft tissue infections where an important part of infections are polymicrobial [14].

De-escalation can be a challenging issue in SICU patients. As discussed above, infections are often present in multiple sites, and are often polymicrobial, limiting the possibilities of de-escalation in these patients. This was found in an earlier study from our center where abdominal infection and the lack of conclusive microbiology were important obstacles to de-escalation [15].

In recent years the changes in the pharmacokinetics in critically ill patients has received increased interest [16]. Although these changes are not limited to surgical patients, again specific issues should be considered in SICU infected patients as it may impact outcome [17]. One of the main determinants of decreased exposure to antimicrobial therapy is increased elimination of the antibiotic (mostly beta-lactam antibiotics) through a phenomenon of augmented renal clearance. Augmented renal clearance is a frequent finding in critically ill patients and certain categories of SICU patients such as trauma and burn patients [18]. In specific patient populations e.g. following abdominal surgery, increased fluid losses though abdominal drains may further decrease antibiotic concentrations [19]. Different dosing strategies such as extended or continuous infusion may be required to improve antibiotic exposure in patients treated with beta-lactam antibiotics [16, 20].

### Source control

Source control frequently is an essential element of the therapy of severe infections in surgical patients. It refers to controlling the source of the infection and includes drainage of pus and inflammatory material as well as debridement of necrotic (infected) tissue. Restoration of anatomy and function is equally important and often these components can be combined in one operation.

Source control should be considered in all patients with severe infections in the SICU. Although the relevance of source control is not limited to SICU patients, the probability that this patient group requires source control is higher due to the high prevalence of abdominal and other surgical infections [1].

As a rule of thumb, source control measures should not be delayed except in situations where demarcation of non-viable tissue and infection is preferable, such as infected pancreatic necrosis, or in situations where source control is difficult to obtain, e.g. an infected driveline of a left ventricular assist device (LVAD). The Surviving Sepsis Campaign (SSC) guidelines suggest that patients should be treated within 12 hours [21], but there is no rationale to defer the intervention unless patient’s physiology is severely impaired and associated with an unacceptable risk of complications during the source control procedure such as coagulopathy or life-threatening metabolic disorders. Comparable to the effect of postponing initiation of antibiotic therapy in case of hypotension, there seems to be a linear increase in mortality when source control is delayed [22]. Timing of source control is often debated and should be guided by the severity of illness (or rapidity of deterioration), the (presumed) source of infection and the physiologic status of the patient (Table 1). Source control interventions may include surgery but also other measures can be – initially – adequate such as PCD or removal of infected tissues or devices.

**Table 1:** **Urgency of source control intervention (after** [23]**)**

Level of urgency	Timing of intervention	Context
**1**	<1-2 h after diagnosis	Rapidly progressive disease e.g. necrotizing fasciitis, intra-abdominal infection with abdominal compartment syndrome
**2**	As soon as patient physiology allows	Limited deferral is acceptable provided antibiotics are administered and patient is not deteriorating e.g. peritonitis
**3**	As soon as infectious process has demarcated	Adequate source control is facilitated and probability of collateral damage lower e.g. infected pancreatic necrosis in a stable patient

Therefore it is crucial that institutions that care for these severely ill patients should have 24/7 access to all diagnostic imaging techniques as well as interventional radiology and surgery. Although practical issues such as operation theatre availability and the lack of expertise are often mentioned as reasons why source control is delayed, there is no scientific evidence that delaying source control is safe, even under broad-spectrum antibiotic coverage. Moreover, in the era of increasing MRD infections, administering antibiotics to patients in whom the source is not controlled could lead to the emergence of antibiotic resistance, or selection of less susceptible microorganisms.

Although once considered a surgical issue, source control measures are no longer limited to the operating theatre. Ultrasound or CT-guided PCD is now an important tool in the early management of severe infections in critically ill patients. Its exact role however remains to be determined, and for many infections a surgical procedure still should be considered the standard of care. PCD however can be a helpful tool during initial resuscitation and correction of metabolic disorders, but also for more difficult to surgically treat infections such as infected pancreatic necrosis, where PCD has emerged as the preferred initial therapy and can effectively avoid surgery in a considerable number of patients [24].

To fully understand the impact of the role of source control in critical care, more attention to this aspect is urgently needed in studies reporting on the outcome of infected patients. Quantifying the residual infection after source control could be helpful to evaluate the role of certain interventions and to guide antibiotic therapy. To this extent we suggest using a classification system (Table 2) that allows to better describe the net effect of source control measures. In analogy with oncological surgery where the R classification reflects the completeness of the surgical procedure, the proposed categorization of residual infection reflects the effect of the source control intervention, supports planning of future treatment and correlates with prognosis. The presence of residual infection refers to the presence of pus, infected tissue after the source control procedure e.g. incomplete drained abscess after PCD or residual necrotic material that cannot be debrided. Ongoing contamination refers to a source that maintains the infection, e.g. a gastrointestinal tract perforation that cannot be transformed into a fistula and continues to soil the abdominal cavity.

**Table 2:** **Source control categorization**

Source control-status	Description
S0	No residual infection
S1	Residual macroscopic infection, no ongoing contamination
S2	Residual macroscopic infection and ongoing contamination

Source control treatment options have been poorly investigated before they entered clinical practice. PCD for example has been studied to some extent in severe acute pancreatitis as part of a step-up minimally invasive approach as opposed to open surgery but for other indications no randomized studies have been performed.

Novel interventions should be subjected to rigorous clinical trials but also treatment strategies that have been taken for granted require re-evaluation. Van Ruler et al. compared the often applied planned relaparotomy approach to a more restrictive on-demand relaparotomy strategy and found the latter to be superior in terms of morbidity and cost [25].

As source control in critically ill is becoming increasingly complex, we advocate the development of multidisciplinary source control teams where intensivists, infectious disease specialists, surgeons and interventional radiologists discuss the need for, the timing of and the preferred methodology used for source control procedures. Continued multidisciplinary evaluation is crucial.

## Conclusions

In conclusion, the management of infections in surgical patients poses specific challenges. Diagnosis is often problematic and the need for source control adds a level of complexity to the management of the patient. Also the need for a multidisciplinary approach can make the decision making difficult. The lack of data have so far led to vague recommendations regarding source control, and clinical studies need to report source control methodology and efficacy. New concepts such as dedicated source control teams may further assist in selecting the most appropriate treatment strategy and further improve outcome of severe sepsis patients in the SICU.
